# MuSeeQ, a novel supervised image analysis tool for the simultaneous phenotyping of the soluble mucilage and seed morphometric parameters

**DOI:** 10.1186/s13007-018-0377-5

**Published:** 2018-12-18

**Authors:** Fabien Miart, Jean-Xavier Fontaine, Christophe Pineau, Hervé Demailly, Brigitte Thomasset, Olivier Van Wuytswinkel, Karine Pageau, François Mesnard

**Affiliations:** 1 Laboratoire de Biologie des Plantes et Innovation, EA-3900, UPJV, UFR des Sciences, 33 rue St Leu, 80039 Amiens, France; 2 Centre de ressources régionales en biologie moléculaire, Bâtiment Serrres-Transfert, rue Dallery, 80039 Amiens Cedex 1, France; 3 Sorbonne Universités, Génie Enzymatique et Cellulaire, UMR CNRS 7025, Université de Technologie de Compiègne, CS 60319, 60203 Compiègne Cedex, France; 4 Present Address: Institut Jean-Pierre Bourgin, UMR1318, INRA/AgroParisTech, Saclay Plant Sciences, INRA Centre de Versailles, 78026 Versailles Cedex, France

**Keywords:** Soluble mucilage, Seed shape, High-throughput phenotyping, Image analysis, Computer-aided, Low-cost, User-friendly, *Linum usitatissimum*, *Arabidopsis thaliana*, Breeding

## Abstract

**Background:**

The mucilage is a model to study the polysaccharide biosynthesis since it is produced in large amounts and composed of complex polymers. In addition, it is of great economic interest for its technical and nutritional value. A fast method for phenotyping the released mucilage and the seed morphometric parameters will be useful for fundamental, food, pharmaceutical and breeding researches. Current strategies to phenotype soluble mucilage are restricted to visual evaluations or are highly time-consuming.

**Results:**

Here, we developed a high-throughput phenotyping method for the simultaneous measurement of the soluble mucilage content released on a gel and the seed morphometric parameters. Within this context, we combined a biochemical assay and an open-source computer-aided image analysis tool, MuSeeQ. The biochemical assay consists in sowing seeds on an agarose medium containing the dye toluidine blue O, which specifically stains the mucilage once it is released on the gel. The second part of MuSeeQ is a macro developed in ImageJ allowing to quickly extract and analyse 11 morphometric data of seeds and their respective released mucilages. As an example, MuSeeQ was applied on a flax recombinant inbred lines population (previously screened for fatty acids content.) and revealed significant correlations between the soluble mucilage shape and the concentration of some fatty acids, e.g. C16:0 and C18:2. Other fatty acids were also found to correlate with the seed shape parameters, e.g. C18:0 and C18:2. MuSeeQ was then showed to be used for the analysis of other myxospermous species, including *Arabidopsis thaliana* and *Camelina sativa*.

**Conclusions:**

MuSeeQ is a low-cost and user-friendly method which may be used by breeders and researchers for phenotyping simultaneously seeds of specific cultivars, natural variants or mutants and their respective soluble mucilage area released on a gel. The script of MuSeeQ and video tutorials are freely available at http://MuSeeQ.free.fr.

**Electronic supplementary material:**

The online version of this article (10.1186/s13007-018-0377-5) contains supplementary material, which is available to authorized users.

## Background

Seeds of many angiosperms, including *Arabidopsis thaliana* and flax (*Linum usitatissimum* L.), become surrounded by a hydrophilic capsule called seed coat mucilage on imbibition [1–4]. This sticky secretion is usually composed of two distinctive parts with a water-soluble outer layer, poorly adherent to the seed surface, and a strongly adherent inner layer [5–8]. Screens for the mucilage content have been widely used in *Arabidopsis* to find out mutants affected in the mucilage biosynthesis and release [9–11]. Current methods were mainly based on visual appreciation of the adherent part of the mucilage, the latter being easily observable because it is tightly attached to the seed coat of Arabidopsis seeds [6]. The soluble part of mucilage is released in all directions and diluted when the seeds are soaked in water. This renders the visual appreciation impossible and the quantification much more complex [9, 12–14]. Access to these mucilage parameters are however crucial to better-understand the ecophysiological role of soluble mucilage layers [6, 16]. Indeed, the majority of the mucilage polysaccharides are contained in this layer [5, 7, 17, 18] and some species release almost only soluble mucilage [9, 15]. It has also been proposed that the soluble mucilage may play a role in the seedling germination capacity [6] and in increasing seed adhesion to soil substrates [1].

Seed coat mucilages are mainly composed of pectic polyssacharides [6, 7, 19, 20]. The classical method to detect seed coat mucilage phenotypes consists in incubating seeds in water containing a specific marker of these pectic polysaccharides such as the Ruthenium Red [5, 7, 21–24]. The dye toluidine blue O is also commonly used to stain the mucilage because of its metachromatic properties [25–27]. Once the seeds soaked, the soluble mucilage disperses in the surrounding medium in all directions and is stained by absorbing the dye. Only major differences can be detected with such a method. A second range of methods consists in the extraction of the soluble mucilage in water. The efficiency of that extraction process is highly variable over a broad range of plant species and varies according to the type of process used [9, 15, 28, 29]. For example, for the *Arabidopsis* reference accession Col-0, the soluble mucilage can be easily recovered by gentle extraction in water. The process of extraction on other natural accessions or mutants often requires the use of dilute chelators such as EDTA or HCL-NaOH [6, 12, 30–33]. Also, a sequential water extraction improves the mucilage extraction efficiency [18, 34, 35], considering a combination of physical parameters such as the time of extraction, the temperature, an ethanol precipitation (or not) and the stirring intensity [18, 34, 36, 37]. Number of mucilage extraction procedures on *Arabidopsis* are summarized, compared and discussed in [9]. Another method consists in measuring a mucilage indicator value (MIV) corresponding to the viscosity of a hot-water extraction of mucilage [38, 39]. Finally, all these water extraction methods are time consuming and particularly hard to apply for the high-throughput quantitative phenotyping of the soluble mucilage content. A mucilage extrusion on agarose plates may be a good strategy for soluble mucilage phenotyping [16]. Since there is any dye in the gel, this is particularly dedicated to the visual evaluation of the released mucilage for the research of mutants showing major differences, but this is not a precise quantitative method. So far none of these methods are suitable for the high-throughput measurement of the soluble mucilage content.

Recent advances in the field of computer-assisted image processing, called bioimage informatics [40], have facilitated the development of a number of image-analysis algorithms, software packages and image-analysis platforms dedicated to the plant morphological traits analysis [41, 42]. Among the wide range of plant species studied with these methods, many of them deal with the seed shape and the seed development [43–47]. Recently, a method for the quantitative analysis of the mucilage area based on image analysis was developed by [48], but it can only be applied for the phenotyping of the adherent part of the mucilage.

Here, we developed a high-throughput phenotyping method called MuSeeQ for the simultaneous extraction of morphometric data from the seeds and their soluble mucilage areas released on the gel. Flaxseed was chosen as a model species for evaluating and validating the method due its high content in mucilage, phenylpropanoids and fatty acids (α-linolenic acid) [28, 38, 49–53]. The first MuSeeQ step allows the uniform release of mucilage around the seed and its staining via the absorption of the dye toluidine blue O contained in the gel. The second step is a digital image analysis tool performing the detection of several seed shape- and soluble mucilage-related traits. Using a combination of image-processing operators, MuSeeQ automatically identifies and segments seeds and soluble mucilages from the agarose stained gel background. It was found good correlations between automated and manual measurements for the area of the soluble mucilage released and for the area of the seeds. The automated procedure is ready to use to extract 11 relevant morphometric parameters related to the seeds and the soluble mucilages. MuSeeQ was next used for studying the carbon partioning between the seedcoat and the embryo. Significant correlations were found between the soluble mucilages morphometric parameters and the content of some fatty acids, i.e. C16:0, C18:2 and the ratio C18:3/C18:2. (Interestingly, some fatty acids were also found to correlate with the seed shape parameters, i.e. C18:0, C18:2 and the ratio C18:3/C18:2.) Finally, MuSeeQ was shown to be usable for other myxospermous species, including *Arabidopsis thaliana* and *Camelina sativa.* To conclude, MuSeeQ represents a novel promising low-cost method for the high-throughtput phenotyping of the soluble mucilage area and the simultaneous measurement of the seed morphometric parameters.

## Results and discussion

### MuSeeQ’s biochemical assay: toluidine blue O staining of the mucilage on a gel

**Fig. 1:**
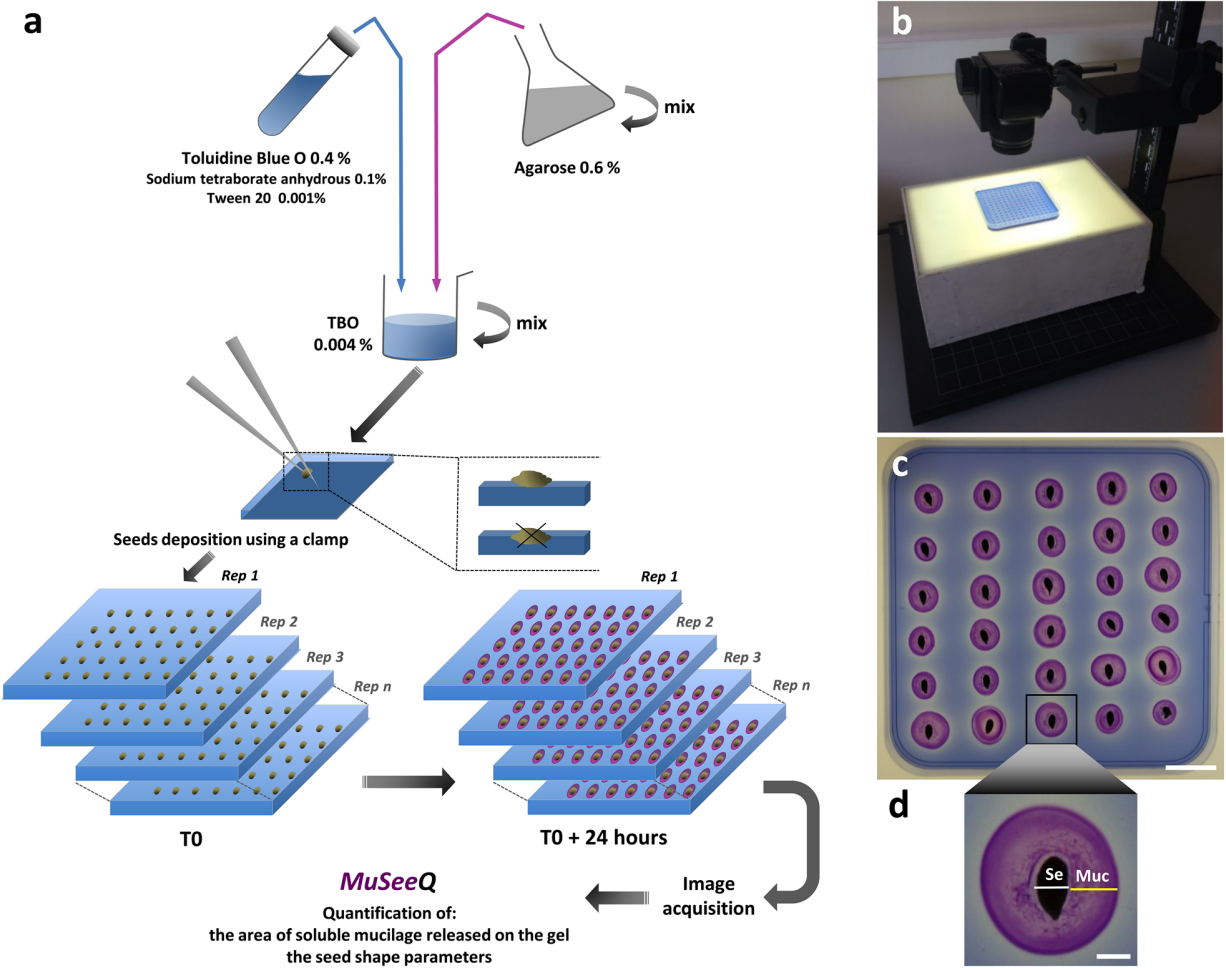
MuSeeQ procedure. **a** Three steps are required: Step 1. The biochemical assay for staining of the agarose gel and for the deposition of seeds. Step 2. Images acquisition. Step 3. A computer-assisted image analysis tool. **b** Experimental setup allowing to take micrographs. The fixed camera mount is placed on top of the agarose gels. **c** Representative image of flaxseeds from the cultivar Oliver and their corresponding soluble mucilages stained with toluidine blue O after 24 h. **d** Zoom on one seed (Se) demonstrating the abilities of the biochemical assay for the release of a soluble mucilage concentric stained halo (Muc). Bars: *C* = 18 mm; *D* = 2 mm

The starting point of our phenotyping method consists of obtaining regular mucilage stained halos (example on flaxseeds: Fig. 1c, d). An agarose gel was chosen as technical support, a method commonly used for imaging root architecture [54, 55]. Almost directly after being deposited on the gel, seeds absorbed the water from the gel and started releasing the mucilage. The dye toluidine blue O was used for staining the translucent mucilage because it turns into a purple pink colour in presence of the pectic mucilage [26]. This stain was more efficient than the dye Ruthenium Red to segment the mucilage from the image background. Seeds have to be carefully deposited on the gel and the released mucilage progressively absorbed the dye toluidine blue O from the gel (Fig. 1a). We experimentally estimated that 24 h were required for a maximum soluble mucilage release and staining efficiency..

### MuSeeQ’s bioimage informatics: the computer-aided image analysis tool

#### Introduction to MuSeeQ’s bioimage informatics

The image-processing program ImageJ [56] was chosen as a platform to host MuSeeQ because it is in the public domain and it is commonly used by the biologist community; ImageJ is presented as the world’s fastest image processing program written in pure Java (https://imagej.nih.gov/ij/features.html). It supports and deals with a wide range of file formats and works on many operating systems. Moreover, the open source platform Fiji (Fiji Is Just ImageJ) [57] provides many powerful image analysis solutions and a curated selection of plugins. MuSeeQ was thus written in the ImageJ’s Java-like macro language to be easily implemented in Fiji as a module. An additional movie file shows how to install MuSeeQ on Fiji [see Additional file 1].

#### Image acquisition and pre-processing

Image capture is a key factor of the accuracy of the high-throughput phenotyping method. To optimize the resolution of the input image, MuSeeQ was applied on digital images of agarose stained gels acquired using a high-resolution flatbed photo scanner [44, 47] or using standard consumer cameras. The image resolution was found to be higher when using stantard consumer cameras, including low-budget imaging setups such as built-in phone cameras. However, high-resolution digital cameras will be preferred in the case of small seeds as it is the case for *Arabidopsis*. The better contrast between the soluble mucilage, the seed and the background, crucial for the segmentation process, was obtained using a fixed camera above the agarose stained gel placed on a light box (Fig. 1b).

**Fig. 2:**
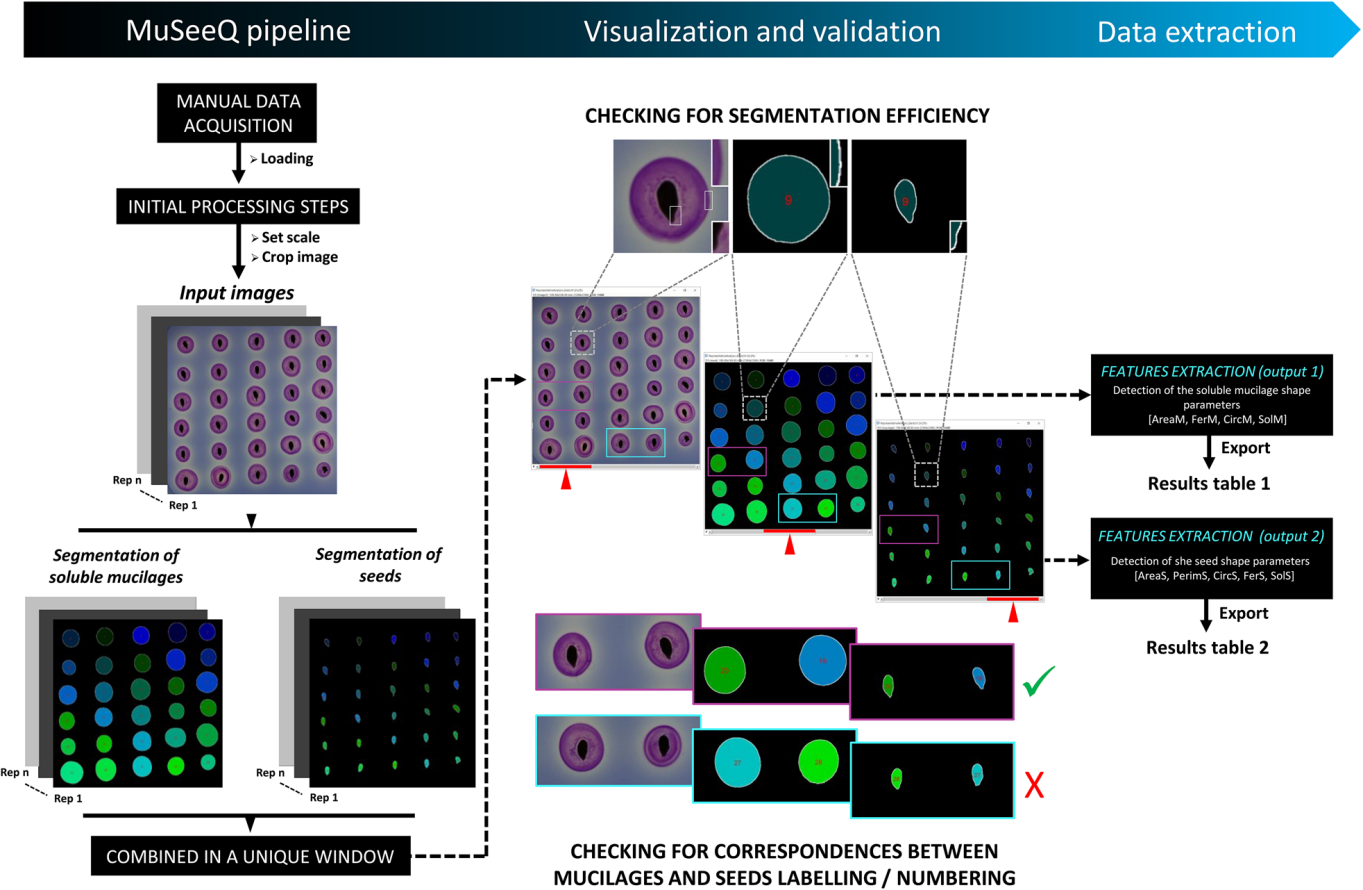
MuSeeQ’s second step: the image analysis process. On the left panel is presented an overview of the MuSeeQ’s workflow. In the middle panel, three screenshots of the final window illustrate how the user can easily visualize and validate the quality of both segmentation processes, simply by playing with the slider (in red) and zooming. From each segmented image are exported all the features describing the soluble mucilages and the morphometric parameters of the seeds (right panel). Please note that only the most relevant parameters are presented here, but others can be computed by MuSeeQ. Bars: 2 mm

The user needs to provide information only at the pre-processing step. Once the digital image is opened and MuSeeQ activated, an exact conversion of the image pixels in the calibrated millimeter unit is required. MuSeeQ requests the user to select both extremities of the agarose stained gel (Fig. 2; Additional file 2). If the dimensions of the square Petri dish are known, the user can directly enter the real dimensions and accept to continue the process. If unknown, the user has to place a graduated ruler next to the Petri dish. The segmentation steps applied by MuSeeQ are based on the identification of objects having a specific pixel colour intensity. To avoid any problems with the edges of the plates, i.e. to detect edges and assimilate them as objects of interest, the user can select a rectangular window within the plate and crop it.

#### Segmentation settings and image processing

It is possible in an 8-bit grayscale image to discriminate two pixels by measuring their difference in term of brightness values ranging from 0 to 255. In colour images, two pixels of distinct colours can have the same brightness. The solution to distinguish pixels with the same brightness consists of expressing images in the RGB 3-D cubic space as a composite of the three primary colours, i.e. red, blue and green. Each colour channel can be independently selected and segmented. However, the HSV colour space (for hue, saturation and brightness value), allows to obtain a better and more natural depiction of colours [58–61]. Although segmentation is not a trivial task for a low signal-to-noise ratio [40], the HSV colour space also demonstrated its higher ability to discriminate plant organs from their background [62–64]. Due to the close colours between flaxseeds (brown to dark brown), soluble mucilages (purple-pink) and the agarose stained gels background (light blue), our algorithm processes input images in the HSV colour space.

Depending on the goal, a number of commonly used methods are available for the segmentation of plant objects in digital images [65], such as noise reduction, skeletonization or thresholding. Threshold-based methods generally require to automatically or manually set fixed threshold levels [66–68]. These methods make irrevocable decisions on the fact that a pixel is part or not of the object of interest and are more likely prone to make errors. To overcome recurrent problems such as an uneven illumination of the image or the background and non-homogenous feature intensities, adaptative threshold solutions have been developed and are freely avaible on Fiji [57]. Here, the threshold is computed locally pixel by pixel based on the image characteristics on a round window with a defined radius. Dynamic approaches also allow to automatically detect image objects whatever the lighting conditions and the monitoring system by fitting a topological or geometrical model of the targeted object to the input data [54, 69, 70]. These top-down methods generally consist of the peak detection into the histogram of all colour components [71, 72], to individualize and segment plant objects showing important differences in terms of contrast, such as soil, leaves, hypocotyls and roots. When working in the HSV colour space, an approximation of the Gaussian curves describing the green (hue) component is sufficient to segment the green parts of plants such as rosettes [64, 73]. In our case, it is impossible to assimilate the soluble mucilage or the seed only to peaks near the hue value component. Consequently, the histograms of hue, brightness and saturation colour components have to be approximated to determine peaks corresponding to each object of interest.

Identification and segmentation tasks may also have been performed using the more and more popular neural networks. This type of machine learning methods provides state-of-the-art performances [74, 75]. Basically, features are computed from raw images and the output of the features is used to extract and separe classes, such as seed morphometric parameters, using a classifier. Among the machine learning methods, Convolutional Neural Networks (CNNs) have become popular for unsupervised classification [76, 77]. Among CNNs, we find Region-based CNNs (R-CNNs) [78] which classifies regions using deep CNN. Nevertheless, this method is very slow to apply. Fast R-CNN was developed to overcome this limitation but the latter is based on a selective search algorithm. Faster R-CNN [79] and R-FCN [80] were also developed to improve the speed and accuracy of the process. While the first method is time-consuming when a large number of proposals is used in its first-stage, R-FCN might be too local to be discriminative enough. Deep machine learning methods have proved nevertheless its performance in image-based plant phenotyping for fully automated quantitative trait identification and localization [75] as well as plant disease detection and diagnosis [80]. Despite the unrivaled accuracy of these techniques, they often require hundreds, sometimes thousands of images and diverse datasets to be trained [81]. Moreover, differences between the image format, size and depth of the dataset used for the training of the network and that used for the analysis can also lead to a misclassification and segmentation troubles for non experts, which led us to avoid CNNs.

In our study, there is no necessity for the power of a top-down thresholding approach or machine learning methods. The algorithm used by MuSeeQ was thought as the combination of the global threshold-based and dynamic segmentation principles. We first modified the G. Landini Colour Threshold function based on an algorithm derived from the iterative intermeans method (IsoData algorithm; [82]). Then, we manually trained our threshold algorithm to a set of digital images. This allowed us to detect peaks in the hue, saturation and brightness value histograms describing as much as possible pixels corresponding to the soluble mucilages and seeds (Additional file 3). Finally, the range of pixel intensity values describing peaks in each of the colour components were computed in our algorithm as default threshold values.

**Fig. 3:**
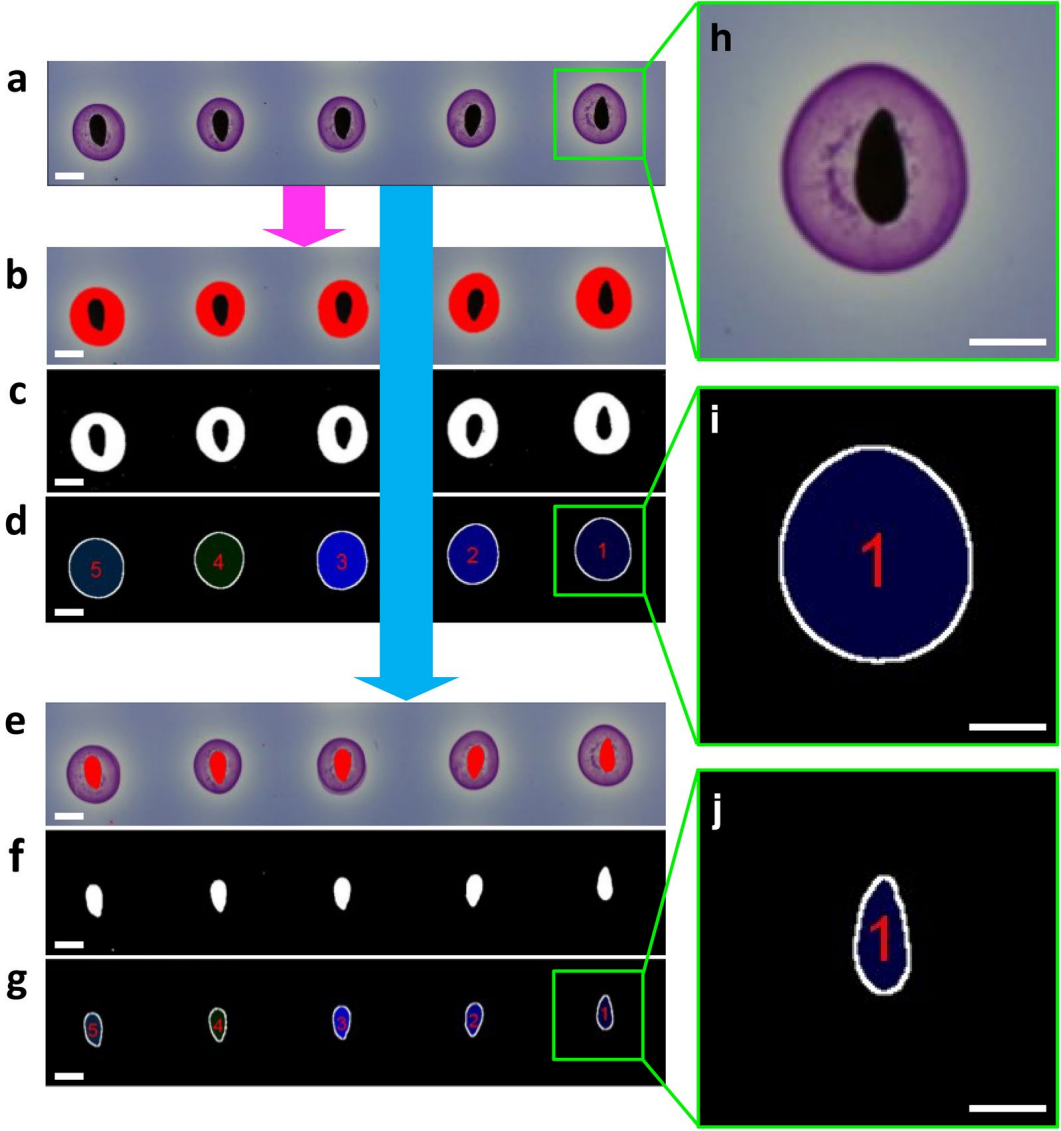
The work flow of the labelling and numbering pipeline. **a** Raw image from the biochemical assay. **b** Initial binary image from the first raw image duplicata after segmentation of the soluble mucilage halos around the seeds (purple-pink). **c** Real area of the soluble mucilages segmented by MuSeeQ including pixels below the seeds. **d** Final step of the segmentation of the soluble mucilages after labelling and numbering. **e** Initial binary image from the second raw image duplicata after segmentation of the seeds. **f** Final step of the segmentation of the seeds after labelling and numbering. **g–i** High-magnification views of one seed and its corresponding soluble mucilage both numbered 28 and stained in light green. Bars: 3 mm

Once digital input images are opened (just by dragging and dropping them in the Fiji toolbar) and scale setting done, the algorithm duplicates the row image (Fig. 2, 3) and processes independently both daughter images. On the first one, MuSeeQ splits the original image in three grayscale channels corresponding to the H, S and V colour components with pixel values ranging from 0 to 255 (Additional file 3). To segment the soluble mucilages, the default threshold values are set to 172, 24 and 85 for H, S and V, respectively (Additional files 3a, 9). If the quality of the threshold is unsatisfactory, the user is asked to refine the range of the default threshold values previously fixed in the tool box. Because the saturation colour component did not influence in a significant way pixels describing the mucilage, we chose to keep only the H and V colour components as manually adjustable parameters (Additional file 2). Segmentation of the soluble mucilage has to consider the entire area of the soluble mucilage (Fig. 3a–c), which comprises the pixels present in the soluble mucilage, between the outline of the external circle and the border of the seed, and the pixels detected in the seed area. This segmented area, AreaM, corresponds to all of the pixels contained inside the perimeter defined by the soluble mucilage outlines (Fig. 3d). Finally, we computed a predetermined range of size for the halo of soluble mucilage, from 18 to 315 mm^2^, which allows to automatically delete two or more soluble mucilages overlapping on the gel. For seed segmentation, MuSeeQ exactly applies the same process on the second image duplicata (Figs. 2, 3e–g). Only the default threshold values, based on the peaks defining each colour component, are modified and set to 255, 255 and 70 for H, S and V, respectively (Additional file 3). At the end of the process, only the threshold values corresponding to the V component can be manually modified by the user to improve the quality of the seed segmentation. Due to the macro format of MuSeeQ, the user can easily modify the default threshold values for the seeds and soluble mucilages (Additional file 4). But to simplify as much as possible the use of our tool for those that are not familiar with Fiji, different versions of the macro are proposed, each one well-suited to five important myxospermous species (*Arabidopsis thaliana*, *Linum usitatissium* L., *Camelina sativa*, *Plantago major* and *Capsella bursa*-*pastoris*), freely available at [83].

#### Segmentation display and validation

MuSeeQ proposes an intuitive way to validate segmentation processes. It assigns a specific number to the soluble mucilage of every analysed seed, allowing the user to easily search the measured parameters in the tables of results (Figs. 2, 3h–j, Additional file 2). The same process is applied in parallel for the seeds. So far, despite the fact that no correlation was found between the area of the seeds and the quantity of soluble mucilage extracted [84], we assumed that the soluble mucilage area may potentially be linked to the seed size and/or the seed shape. We decided to normalize the area of each soluble mucilage by the area of their respective seed (AreaS). The quantification of the soluble mucilage area was assimilated to the ratio (AreaM/AreaS), i.e. the « adjusted mucilage content » . Combining both kinds of data in order to compute this ratio can not be made automatically by MuSeeQ. To do so, the easiest way consists in using the labelling and numbering of the segmented seeds and soluble mucilages. MuSeeQ attributes colours and numbers to seeds and mucilages line by line from the top to the bottom of the image. For a perfect matching between seeds and mucilages, seeds have to be sown on the agarose gel according to a diagonal. Thus, the uppermost seed and its corresponding mucilage will be numbered “one” and the lowest seed and its mucilage will be associated to the last number. Therefore, if seeds of the same size do not release the same quantity of soluble mucilage, the soluble mucilages with the biggest areas may be detected first by MuSeeQ, which would lead to a mismatch between seeds and mucilages numbers and colours. For example, seed no. 28 corresponds to mucilage no. 27 (Fig. 2). To solve this problem and because it is not necessarily straight-forward for a beginner to deposit the seeds properly on the medium, we propose several templates for the seed deposition of three major species (Additional file 5).

Since threshold pixels corresponding to the seeds and their mucilages overlap (Fig. 3a–i), another contribution of this work for the validation of the segmentation results was to display them separately. Once the segmentation steps finished, the three screens corresponding to the input image, the segmented mucilages and the segmented seeds are combined together in a unique window (Fig. 2). For checking the quality of both segmentations as well as the correct matching between labels, the user is finally asked to zoom on the objects of interest and play with the slider to move among the three images (Fig. 2, Additional file 2).

### Extraction of the parameters of interest

The measurements performed by MuSeeQ are extracted from input images and exported in two tables of results, either for the soluble mucilage- or seed-related parameters (Fig. 2). MuSeeQ computes eleven mathematical shape descriptors. Five of the most relevant, both for the seed and the soluble mucilage, are summarized in Table 1 with their respective unit, description and abbreviation. Regarding the mucilage, the most important one corresponds to the area of the surface of released soluble mucilage in 2D (AreaM). MuSeeQ also computes the area of the seeds surface projected in 2D (AreaS), a classical way to access the seed size [44, 47].

**Table 1:** Overview of the main soluble mucilage- and seed shape-releated parameters computed by MuSeeQ

Traits	MuSeeQ designation	Description	Code
Seed	Area (mm^2^)	The area of the seed surface projected in 2D. Computed as: AreaS = π × radius^2^	AreaS
	Circularity	Corresponds to a more or less elongated seed shape. Circularity is comprised between 0.0 and 1.0. A value of 1.0 indicates a perfectly round seed. Computed as: CircS = 4π(AreaS/PeriS^2^)	CircS
	Feret (mm)	The longest distance between two points of the segmented seed. For seeds, it can be interpreted as the longest distance between the pedoncule and the awn	FerS
	Perimeter (mm)	The length of the limit of the segmented seed. Computed as: PeriS = 2 × π × radius	PeriS
	Solidity	Corresponds to the ratio between the exact and the approximated seed area, using a sort of rubber band all around the segmented seed. Computed as: SolS = AreaS/Convex AreaS	SolS
Soluble Mucilage	Area (mm^2^)	The area of the surface of released soluble mucilage projected in 2D. Computed as: AreaM = π × radius^2^	AreaM
	Circularity	Corresponds to a more or less elongated mucilage shape. Circularity is comprised between 0.0 and 1.0. A value of 1.0 indicates a perfectly round mucilage. Computed as: CircM = 4π(AreaM/PeriM^2^)	CircM
	Perimeter (mm)	The length of the limit of the segmented mucilage. Computed as: PeriM = 2 × π × radius	PeriM
	Solidity	Corresponds to the ratio between the exact and approximated mucilage areas using a sort of rubber band all around the segmented mucilage. Computed as: SolM = AreaM/Convex AreaM	SolM
Ajusted mucilage content		The 2D surface of released mucilage corrected by its corresponding seed surface projected in 2D	Ratio (AreaM/AreaS)

Please note that the number of parameters which could be analysed can be increased using other plugins and functions in ImageJ

Interest in computing our algorithm under the Java-like macro language resides in its flexibility, which implies that the user can easily increase the number of parameters to analyse by simply adding them into the script (Additional file 6). MuSeeQ can also be complemented by a high number of plugins and macros in Fiji such as editing, colour processing, measures or image enhancement [85, 86].

### Statistical validation

**Fig. 4:**
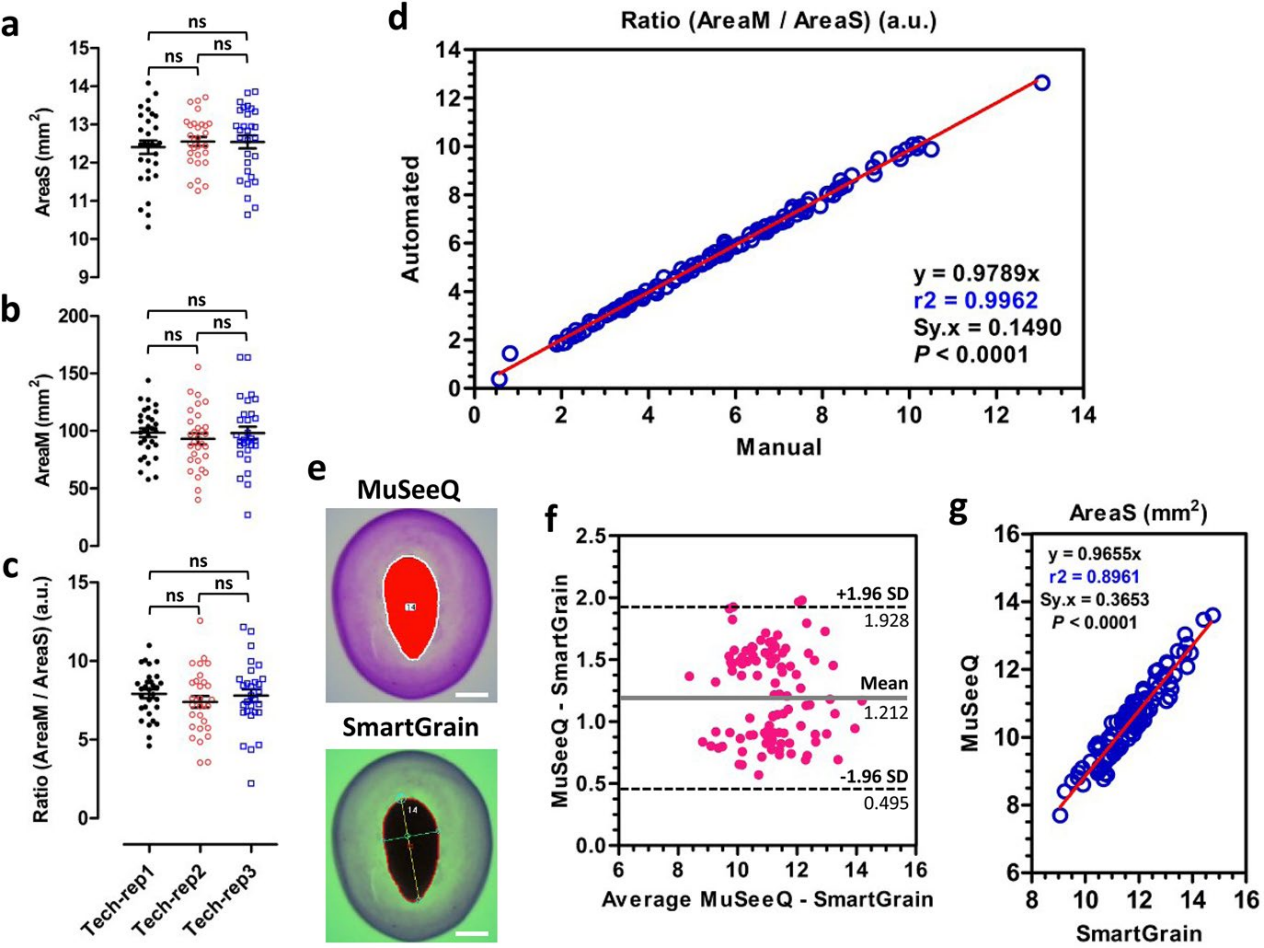
Statistical validation of MuSeeQ. **a–c** Technical reproducibility assays for the measurement of the area of the seeds (**a**), the area of released soluble mucilage (**b**) and the ratio (AreaM/AreaS) (**c**) on seeds from the cultivar Oliver sown on three different agarose stained gels. ns indicate no significant differences (*p* ≤ 0.05) as determined by one-way ANOVA followed by a Tukey’s HSD (honest significant difference) post hoc test (n = 30 seeds for each technical replicat). Bars represent mean ± SEM **d** Correlation analysis of automated (MuSeeQ) versus manual (Fiji) measurements of the ratio (AreaM/AreaS) (n = 104 seeds from 21 randomly selected flax RILs, 5 seeds each). **e** A representative result of segmentation using MuSeeQ and SmartGrain on the same seed. Note that both image analysis tools numbered the seed 14. Opposite to SmartGrain, MuSeeQ does not draw the computed parameters on the seed, except for the seed outlines. **f** Bland–Altman plot showing no major statistical differences between the results of AreaS obtained using both methods. **g** Correlation analysis of automated (MuSeeQ) versus manual (SmartGrain) measurements of the AreaS. Pearson’s correlation coefficients were determined by linear regression analyses (n = 104 seeds from 21 randomly selected flax RILs, 5 seeds each). Red lines are least squares regression curves. Bars: 1.5 mm

First, we checked the technical reproducibility of the biochemical assay. Seeds from the cultivar Oliver were sown on three different biochemical assays. No statistical difference was found between the technical replicates for AreaM, AreaS and the ratio (AreaM/AreaS) (Fig. 4a–c). In order to statistically demonstrate the efficiency of MuSeeQ for the precise automated segmentation of the mucilages and seeds, the most relevant parameters, i.e. AreaM, AreaS, and the ratio (AreaM/AreaS), were measured on seeds of 21 randomly selected recombinant inbred lines (RILs) from a cross between the cultivars Oliver and Viking using MuSeeQ, and compared to manual measurements using Fiji. The Pearson’s product-moment correlation analyses between automated (MuSeeQ) and manual (Fiji) measurements have demonstrated strong correlations for AreaM (r_2_ = 0.9970; *P* < 0.0001), AreaS (_r2_ = 0.9743; *P* < 0.0001) (Additional file 7), and the ratio (AreaM/AreaS) (r_2_ = 0.9962; *P* < 0.0001) (Fig. 4d).

It is currently not possible to statistically compare our results obtained on the area of the soluble mucilage with other image-based analysis methods since they do not exist yet. However, SmartGrain is an image analysis software dedicated to the quantification of the seed shape parameters [45]. To compare the results obtained with MuSeeQ versus SmartGrain, we applied a Bland–Altman comparison method on the most important seed shape parameter, i.e. AreaS, which confirms the precision of the measurements using MuSeeQ (Fig. 4f). The measurements performed with MuSeeQ slightly exceed those obtained with SmartGrain. This bias is explained by the scale setting step which can slighly vary between the two softwares. Nevertheless, a Pearson’s product-moment correlation analysis

$$r = \frac{N\sum xy - (\sum x)(\sum y)}{{\sqrt {[N\sum x^{2} - } (\sum x)^{2} ][N\sum y^{2} - (\sum y)^{2} ]}}$$

on the same dataset reveals a highly acceptable r_2_ (r_2_ = 0.8961; *P* < 0.001) (Fig. 4g).

### Evaluation of the technical efficiency

**Fig. 5:**
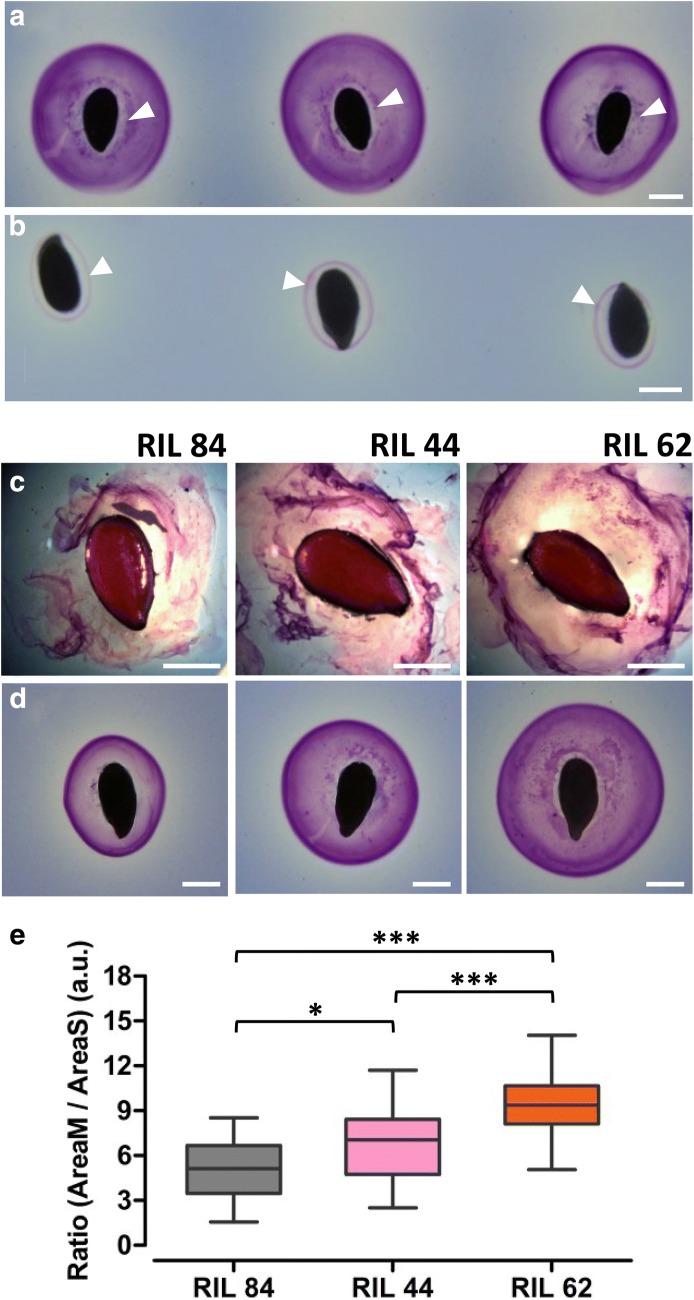
MuSeeQ allows to quantitatively discriminate between close soluble mucilage phenotypes. Pictures showing flaxseeds sown on (**a**) a biochemical assay directly or (**b**) after 24 h of soluble mucilage extraction in water. In both cases, pictures have been taken after 24 h of mucilage release on the gels. White arrows indicate that the seeds are capable of releasing the adherent part of the mucilage on the gel (**b**). **c** Soluble mucilage phenotyping in water containing toluidine blue O after 24 h of release. **d** Soluble mucilage phenotyping using the MuSeeQ’s biochemical assay after 24 h of release. Seeds in (**c**) and (**d**) were chosen as the most representative of the corresponding RILs. **e** Statistical analysis of the ratio (AreaM/AreaS) showing different phenotypes among the three RILs. Significant differences were determined using a one-way ANOVA followed by a Tukey’s HSD post hoc test, *p < 0.05; ***p < 0.001 (n > 21 seeds for each RIL). Bars: 2 mm

When flaxseeds are placed on a biochemical assay both the soluble and adherent parts of the mucilage seem to be released on the gel (Fig. 5a). To check it, flaxseeds were immersed in water for 24 h in order to extract the soluble part of the mucilage [7, 84] and seeds were then placed on a biochemical assay. Since a thin halo of mucilage is still detected arround the seed (Fig. 5b), this means that our biochemical assay does not allow the full release of the adherent part of the mucilage. However, we assume that it can be used as a high-throughput phenotyping of the soluble mucilage of flaxseeds.

To validate the efficiency of MuSeeQ to perform precise phenotyping of the soluble mucilage, we chose to apply our tool on three randomly selected flax RILs, i.e. the RILs 84, 44 and 62. The soluble mucilage content was visually accessed using the toluidine blue O water staining method [26]. Here, seeds are directly soaked in water containing the dye toluidine blue O. The soluble mucilage quickly diffuses in all directions and is stained by absorbing the dye. Differences in the phenotypes between the three RILs were not clearly obvious, with « soluble mucilage strips » more or less thick depending on the RILs, and were impossible to quantify (Fig. 5c). Using the MuSeeQ’s biochemical assay, differences between RILs became clearer (Fig. 5d). Applying our image analysis tool, we were able to measure and statistically discriminate RILs from each other (Fig. 5e).

Another goal of our phenotyping tool consists of considerably reducing the time of analysis. We estimate that MuSeeQ allows to analyse the soluble mucilage area at least 40 times faster compared with a mucilage extraction in water. Compared with SmartGrain [45], the seed shape parameters analysis takes the same time.

### MuSeeQ, a tool to better-understand the seed physiology

**Fig. 6:**
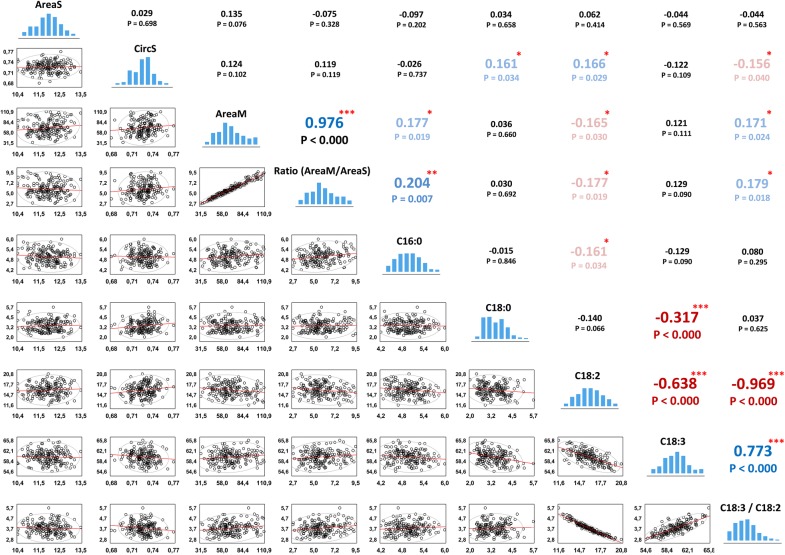
Correlation matrix between traits linked to the soluble mucilage, seed shape and fatty acids. Only pairwise correlations between the most relevant parameters were displayed in the matrix. Correlations between other traits can be found in Additional file 3. The datasets used to build the matrix were obtained using MuSeeQ for the seed shape- and the soluble mucilage-releated traits (10 biological replicates) and from a GC-FID analysis for the FA content (2 biological replicates), both performed on the same recombinant inbred lines population in generation F8. Below the diagonal of histograms are displayed the scatterplots of each pairwise correlation. Values above the diagonal represent the Pearson’s product-moment correlation coefficients (*r*). The negative correlations are depicted in red while positive correlations appear in blue. The colour intensity follows the strength of the correlation. *, ** and *** asterisks indicate significant difference with *p* < 0.05, *p* < 0.01 and *p* < 0.001, respectively

It has been proposed in *Arabidopsis* that during the seed development the main carbon source from photosynthesis could be shared between the seed coat, for the mucilage polysaccharides biosynthesis, and the embryo, for the oil biosynthesis [87]. However, functional studies of *tt2* mutants have shown that a significant reduction of the seed oil content in the embryo and a modification of the fatty acids (FA) composition do not affect the seed coat mucilage production [88]. Other studies on flax have also revealed that the mucilage would not be correlated with the oil and protein contents [39, 89]. This suggests disconnected and/or parallel regulatory pathways for the mucilage and oil biosynthesis, and emphasizes the need for a better understanding of these complex mechanisms. In this context, MuSeeQ was applied on a RILs population in F8 derived from the cross between the cultivars Oliver and Viking in order to detect putative correlations between seed shape- and soluble mucilage-related traits. At least, eleven shape descriptors were computed on the seeds and the mucilages (Additional file 2), but the most important are shown in Fig. 6. The RILs population was screened in parallel for its FA content. The oil content and sixteen FAs ratios were also added to the analysis since the ratio omega-3 to omega-6, i.e. C18:3/C18:2, has been found to play an important role in human health by preventing many diseases [90, 91]. In general, the Pearson’s product-moment correlation coefficients observed are small (Fig. 6; Additional files 8, 9).

It has been shown that the soluble mucilage content could be linked to the seed shape in *Arabidopis* [92–96]. Looking at the correlations matrix between seed shape parameters and other traits, the solidity of the seed (SolS), which indicates how curved the borders of the seeds are (Table 1), was found to positively correlate with AreaM and the ratio (AreaM/AreaS) (Additional files 8, 9). It suggests that the more flaxseed release soluble mucilage, the less flaxseed present curvarures on both sides. Among FAs, C18:0, C18:2, C18:3 and several FAs ratios linked to C18:0 and C18:2, e.g. C18:3/C18:2, correlate with both CircS and SolS (Fig. 6; Additional files 8, 9). These results point out physiological connections between the seed morphometric parameters and the FAs content. On the other hand, the major part of the correlations found between each FA species were significantly negatives (Fig. 6; Additional files 8, 9), which confirms the results obtained on three years and two megaenvironments study of 390 flax accessions and 243 RILs [53, 97, 98].

Even it has been reported on flaxseed that the polysaccharides mucilage content does not correlate with the seed oil content [89], recent studies on *Arabidopsis* demonstrated negative correlations between them [87]. These works have been performed on the total seed oil content, without considering each FA species independently. Our pairwise correlation analyses revealed that AreaM and the ratio (AreaM/AreaS) positively correlate with the C16:0 content (*P* < 0.05 and *P* < 0.01, respectively) and the ratio (C18:3/C18:2) (*P* < 0.05), but negatively correlate with the C18:2 content (*P* < 0.05) (Fig. 6; Additional files 8, 9). Our results suggest that the relationships between the mucilage and the oil biosynthesis pathways could be more complex than as previously described [39, 87, 89]. They also suggest that the released mucilage content could be more likely linked to the FAs composition instead of the seed oil content. Finally, we cannot omit that the correlations were relatively low, which suggests a complex regulation of the mucilage biosynthesis pathway by other seed traits.

### Use of MuSeeQ on various plant species

**Fig. 7:**
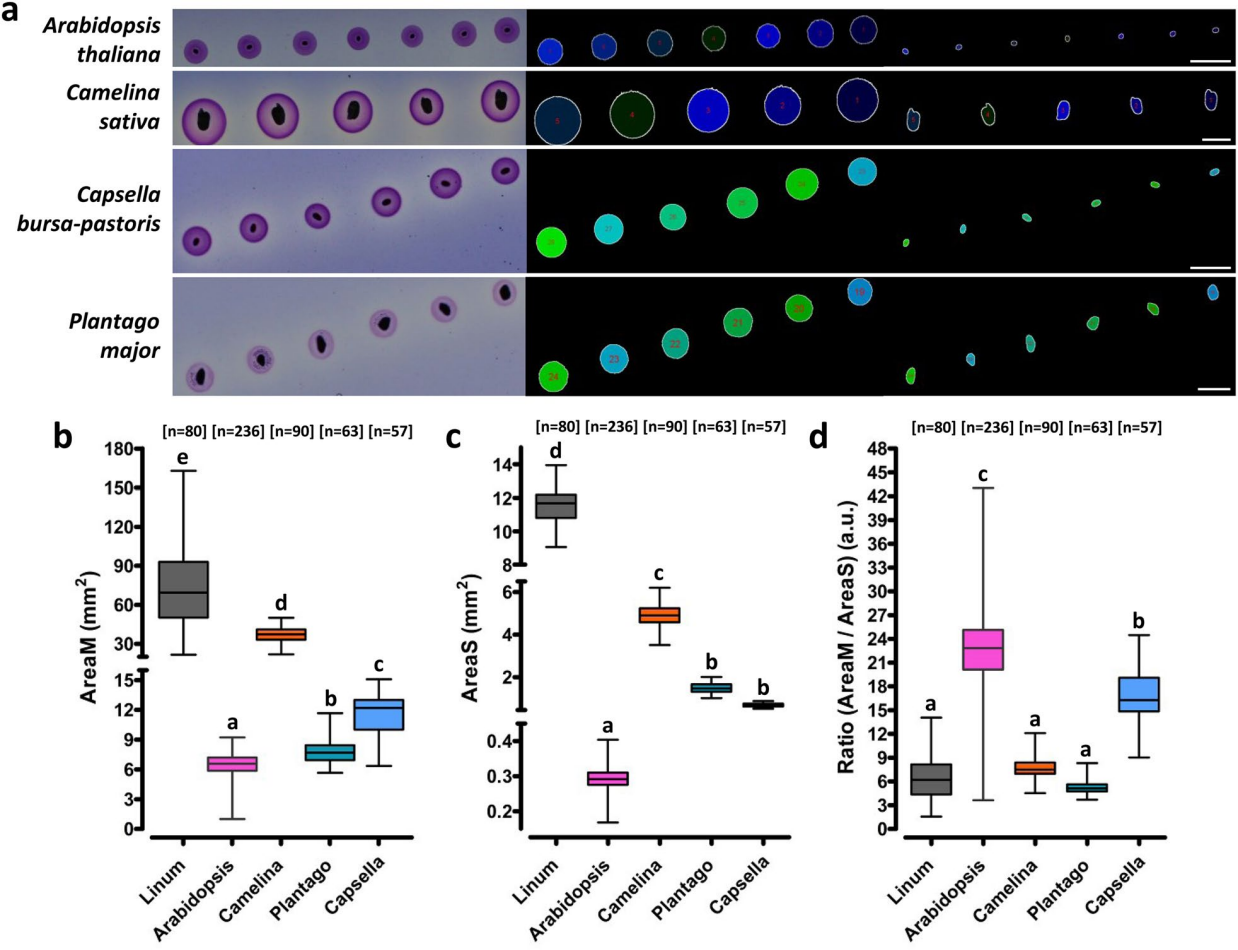
Application of MuSeeQ on other fundamental and crop species. **a** For each species are presented, from the left to the right, the input images, images corresponding to the segmented soluble mucilage and those corresponding to the segmented seeds. Note that the number of seeds and soluble mucilages which can be analysed on a single line on an agarose plate vary across species. We provide a specific version of MuSeeQ for each of these four species. Comparative phenotypic analyses between the three most relevant species for (**b**) the area of the seeds, (**c**) the area of the soluble mucilages and (**d**) the ratio (AreaM/AreaS). Significant differences were determined using a Kruskal–Wallis non-parametric test followed by a Dunn’s multiple comparison post hoc test (n: number of phenotyped seeds). One biochemical assay (one plate) was used for each species except for *Linum* for which we combined the results from three biochemical assays to homogenize the quantity of collected data between species. Bars: 2 mm

We also tested the flexibility of MuSeeQ by applying it on four others important myxospermous species (Fig. 7). The ease of use of our tool and the time required per analysis were the same for all the species, despite the great difference in the number of seeds that can be analysed by plate (Linum: ~ 30; Arabidopsis: ~ 200; Camelina: ~ 80; Plantago: ~ 60; Capsella: ~ 60). MuSeeQ efficiently detected and segmented mucilages and seeds (Fig. 7a). Our quantitative analysis confirmed that each species released different amounts of soluble mucilage (Fig. 7b). We also found huge differences between the species for AreaS (Fig. 7c). When AreaM was corrected by AreaS, the differences observed between species were considerably reduced (Fig. 7d). Thus, MuSeeQ can be used to screen the seeds and soluble mucilages of various species.

## Conclusions

Whereas current methods used to access the soluble mucilage content are time consuming, MuSeeQ not only enables its high-throughput phenotyping for any myxospermous species, but also the simultaneous measurement of the seed shape morphometric parameters in a fast and semi-automated way.

In order to help breeders and researchers to incorporate MuSeeQ in their phenotyping strategies, genetic and functional analyses, it has been developed as a user-friendly tool. It is freely distributed to the scientific community and can be downloaded on the dedicated website (http://MuSeeQ.free.fr) [83].

## Methods

### Plant material and growth conditions

Breeder seeds of *Linum usitatissimum* L. cv Oliver, a winter linseed and Viking, a spring fiber flax and 174 recombinant inbred lines (RILs) from the cross of these two varieties were kindly provided by INRA Centre de Versailles, France. Seeds were selfed in a greenhouse up to F8 under the following conditions: 60% humidity, 21 °C/15 °C day/night regime and with a 16-h photoperiod and were then multiplied in the field and used for the phenotyping of both the mucilage and FAs contents. Arabidopsis seeds (*Arabidopsis thaliana*, Columbia-0 accession) and *Camelina sativa* were from seed stocks propagated in our laboratory. *Plantago major* and *Capsella bursa*-*pastoris* seeds were kindly provided by Professor Michael Deyholos (The University of British Columbia IK Barber School of Arts & Sciences – Canada).

### Soluble mucilage staining

The procedure required for the soluble mucilage staining on the agarose plates, i.e. the MuSeeQ’s biochemical assay, is detailed in the main text, in Fig. 1 and in Additional file 10.

Validation of the technical reproducibility of MuSeeQ (Fig. 4a-c) was done using three replicates on different biochemical assays, 29 seeds of the cv Oliver for each. For the statistical validation of MuSeeQ (Fig. 4, Additional file 7), the measurements of AreaM, AreaS and the ratio (AreaM/AreaS) were performed on 21 randomly selected RILs, five seeds each. For determination of the capacities of the seeds to release the soluble and a part of the adherent mucilage [see Fig. 5a, b], flaxseeds were directly sown on the gel or after 24 h of soluble mucilage extraction in water, and MuSeeQ was applied on digital images of the gels. Analysis of the mucilage release for the RILs 84, 44 and 62 was performed by placing seeds in water containing 0.01% toluidine blue O (Merk Millipore) without shaking. After 1 h seeds were observed using a light microscope (Axioplan 2, Zeiss, http://www.zeiss.fr/). For the soluble mucilages phenotyping of the RIL population (Fig. 6, Additional files 8, 9), we analysed five seeds per RIL and six RILs per assay. We performed two technical replicates and the results were combined together. Finally, the dataset used in Fig. 7 is from experiments performed with MuSeeQ on one biochemical assay per species, except for flaxseeds where we combined the analyses of three different biochemical assays to increase the size of the dataset (Additional files 11, 12).

### FA phenotyping

Analysis of the FA composition was performed on mature dry seeds from all the RILs in F8. Seed oil extraction was performed on 100 mg of fresh dried seeds. Seeds were ground in liquid nitrogen. One mL of diethyl ether was added to the samples, mixed at 650 rpm for 5 min and centrifuged at 8000 rpm for 2 min. The supernatant was collected and ether evaporated. For FA composition analysis, 5 µl of n-hexane (Fisher) containing 0.005% (w/v) pentadecane were added to the pellet. Fifty µl of TMAH (TétraMéthyl Ammonium Hydroxide) were added to the samples. Samples were incubated at 20 °C at 990 rpm for 10 min and were centrifuged at 12,000 rpm at 25 °C for 10 min. One hundred fifty µl of supernatant were transferred in a glass vial. Two biological replicats were considered for each RIL of the population.

FA methyl esters analysis was performed on a TRACE GC ULTRA system coupled with a DSQ II quadrupole mass spectrometer (Thermo Scientific, France), equipped with an automated sample injector. For each sample, 1 µl was injected. The injection was performed with a 25:1 split ratio at 240 °C. For this step, the ion source was adjusted to 220 °C and the transfer line to 280 °C. For analysis, we selected the electron-impact ionization method (EI, 70 eV). The carrier gas used was Helium, with a flow rate of 1 mL min-1. Separation of FA methyl esters was performed using a polar column (length: 60 m, inner diameter: 0.25 mm, 0.25 µm liquid membrane thickness, TR-FAME, Thermo Scientific, France) at 150 °C for 1 min. A gradient of 10 °C min^−1^ was used to increase the temperature to 180 °C and the temperature maintained at 180 °C for 30 s, ramped at 1.5 °C min^−1^ to 220 °C, 30 s at 220 °C, followed by 30 °C min^−1^ to reach 250 °C and maintained at 250 °C for 5 min (run time: 38 min). Each mass spectrum was recorded with a scanning range of 50 to 800 m/z. X Calibur software (Thermo Scientific) was used for manual peak integration and analysis. Each FA species was identified according to its retention time by comparing it with those of pure standards (Fame mix C14-C22 Supelco-18917-1AMP). FA and standards were analysed under the same conditions. Pentadecane was applied as the internal standard. FA species concentrations for each sample were normalized against the internal control and expressed as a pourcentage of the total FA content.

### Statistical analyses and data evaluation

In order to validate flaxseeds segmentation using MuSeeQ, we used SmartGrain software [45]. Linear regression analyses, scatter dot plots, Box and Whiskers plots and the Bland–Altman plot were carried out using GraphPad Prism (Version 5.0, GraphPad Sofware, Inc.). For linear regression analyses, the standard deviation of the residuals was computed to estimate the goodness-of-fit as:

$$S_{y.x} = \sqrt {\sum (residual^{2} /\left( {n - K} \right)}$$

Frequency histograms were made using Matlab software (2014b, The Matworks Inc, Natick, Massachusetts, USA). After having checked that the variables were normaly distributed, Pearson’s correlation analyses were performed using Statistica software v.9.1 (StatSoft, Inc., 1984–2010). The Pearson’s correlation matrix and the corresponding likelihood matrix were created using Statistica (Additional files 8, 9) or after importation of the data into Matlab software (Fig. 6).

For all statistical analyses, the normality and the homocedasticity of the datasets were checked using D’Agostino & Pearson omnibus normality and Bartlett’s tests, respectively. Statistical differences between samples were tested using either a one-way ANOVA followed by a Tukey’s HSD (honest significant difference) post hoc test (Fig. 4, 5) or a Kruskal–Wallis non-parametric test followed by a Dunn’s multiple comparison post hoc test (Fig. 7), with *p < 0.05; **p < 0.01; ***p < 0.001.

## Additional files

**Additional file 1.** Demo video showing how to install the different versions of MuSeeQ in Fiji.

**Additional file 2.** Demo video showing how to use MuSeeQ to analyse your datasets.

**Additional file 3.** Principle of the thresholding method computed by MuSeeQ. The method is used here to dynamically detect peaks in all colour components of the HSV colour space. Spectrum areas highlighted in yellow correspond to the pixels describing as precisely as possible the soluble mucilages (left panel) and the seeds (right panel). **a** Only hue and brightness colour components are important to detect soluble mucilages. Pixels from 24 to 255 on the saturation channel were selected for minimizing the remaining background noise. **b** The brightness (value) colour component alone is important for the segmentation of the seeds.

**Additional file 4.** Demo video showing how to modify the script to use MuSeeQ on other species.

**Additional file 5.** Supports that can be used to help for the seeds sowing on the biochemical assays for *Linum usitatissimum* L.

**Additional file 6.** Supports that can be used to help for the seeds sowing on the biochemical assays for *Camelina sativa*.

**Additional file 7.** Supports that can be used to help for the seeds sowing on the biochemical assays for *Arabidopsis thaliana*.

**Additional file 8.** The script of the MuSeeQ macro.

**Additional file 9.** Correlation analysis of automated (MuSeeQ) versus manual (Fiji) measurements for (**a**) the area of the surface of soluble mucilage released in 2D (AreaM) and (**b**) the area of the seeds surface projected in 2D (AreaS). Since data are sampled from Gaussian populations (D’Agostino & Pearson omnibus normality test), Pearson’s correlation coefficients were determined by linear regression analyses on n = 104 seeds from twenty RILs and nine agarose stained gels.

**Additional file 10.** Pearson’s correlation matrix between the soluble mucilage, seed shape parameters and FAs-releated traits. The negative correlations are depict in red whereas positive correlations are in blue. The colour intensity follows the strength of the correlation.

**Additional file 11.** Likelihood matrix corresponding to the Pearson’s correlation matrix between the soluble mucilage, seed shape parameters and fatty acids-releated traits. The negative correlations are depict in red whereas positive correlations are in blue. The colour intensity follows the strength of the correlation.

**Additional file 12.** Protocol for the preparation of the biochemical assay.

## Abbreviations

C16:0: palmitic acid
C18:0: stearic acid
C18:1: oleic acid
C18:2: linoleic acid
C18:3: α-linolenic acid
EDTA: ethylenediaminetetraacetic acid
HCL: hydrochloric acid
NaOH: sodium hydroxide
MIV: mucilage indicator value
RGB: red, green, blue
HSV: hue, saturation, value
EM: expectation maximization
2D: two dimensions
3D: three dimensions
QTL: quantitative trait loci
TMAH: tétraméthyl ammonium hydroxide
RIL: recombinant inbred ines
FA: fatty acids
ANOVA: analysis of variance
GC-FID: gaz chromatography-flame ionization detector
RIL: Recombinant inbred lines
mm: millimeter
