# Supplementary figures and images for: MuSeeQ, a novel supervised image analysis tool for the simultaneous phenotyping of the soluble mucilage and seed morphometric parameters
Source: Plant Methods. 2018 Dec 18;14:112. doi: 10.1186/s13007-018-0377-5 (PMC6297999; doi:10.1186/s13007-018-0377-5)

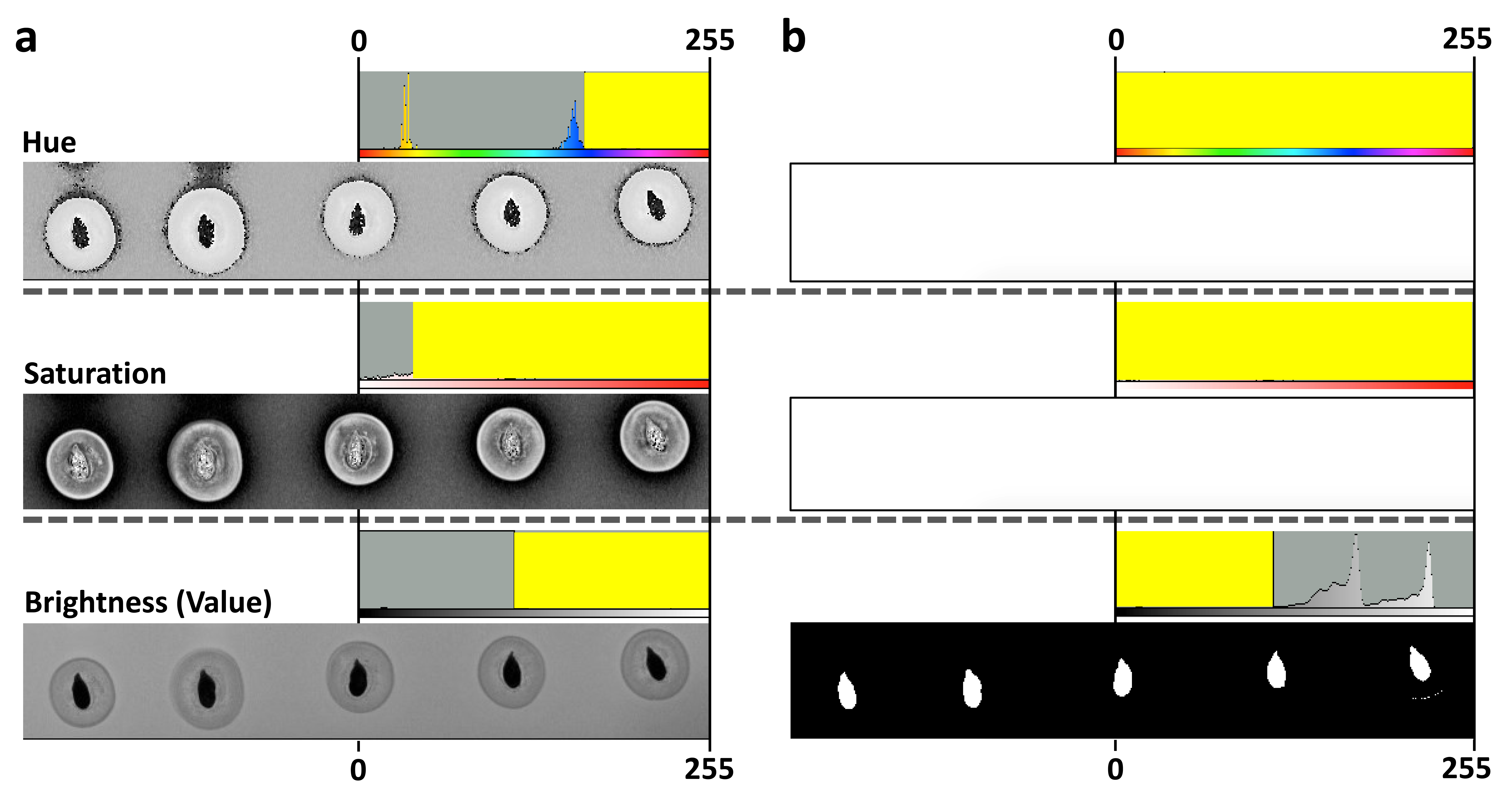

Supplement: Supplementary file 3 — Additional file 3. Principle of the thresholding method computed by MuSeeQ. The method is used here to dynamically detect peaks in all colour components of the HSV colour space. Spectrum areas highlighted in yellow correspond to the pixels describing as precisely as possible the soluble mucilages (left panel) and the seeds (right panel). a Only hue and brightness colour components are important to detect soluble mucilages. Pixels from 24 to 255 on the saturation channel were selected for minimizing the remaining background noise. b The brightness (value) colour component alone is important for the segmentation of the seeds. [file 13007_2018_377_MOESM3_ESM.jpeg]

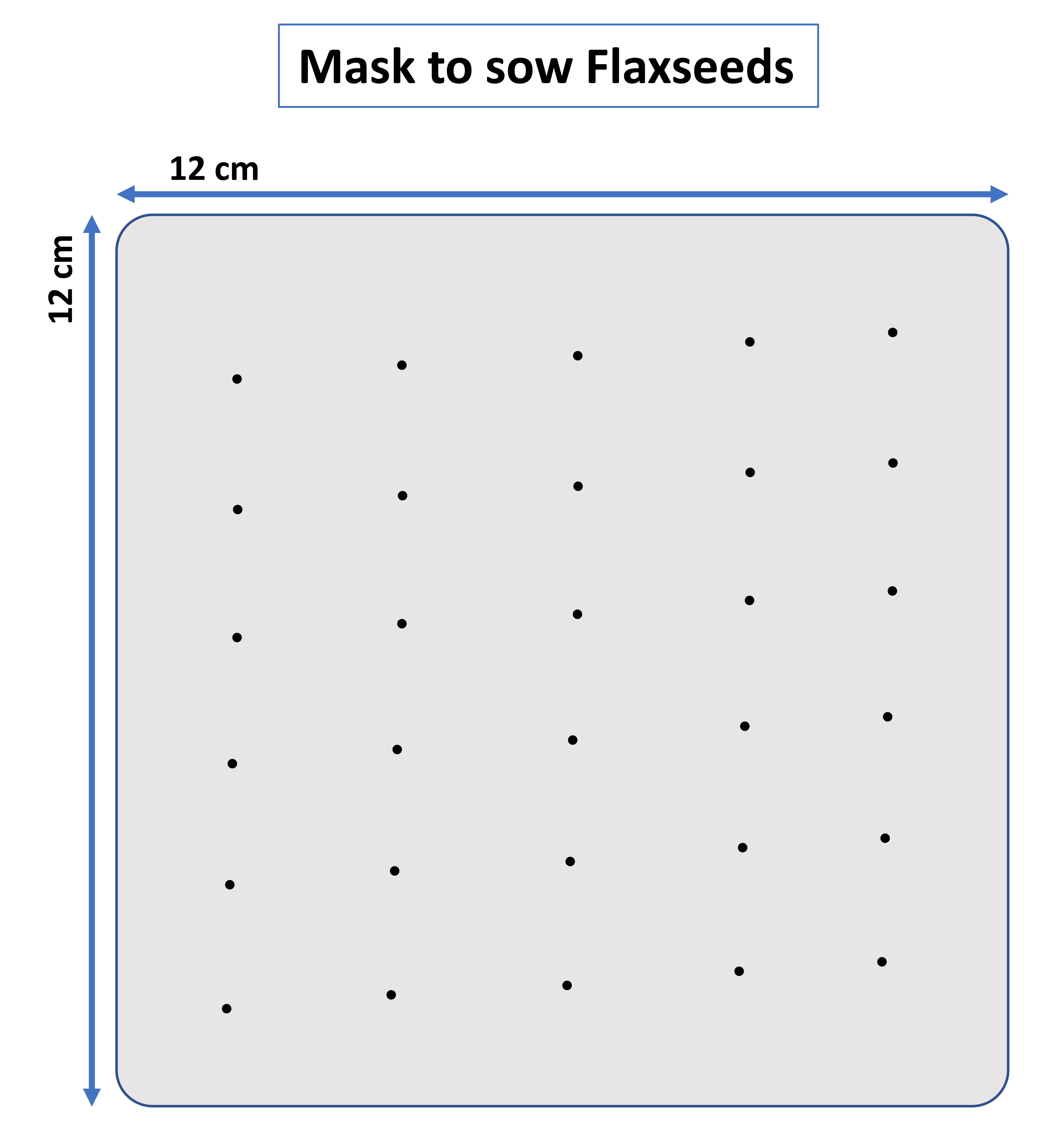

Supplement: Supplementary file 5 — Additional file 5. Supports that can be used to help for the seeds sowing on the biochemical assays for Linum usitatissimum L. [file 13007_2018_377_MOESM5_ESM.jpg]

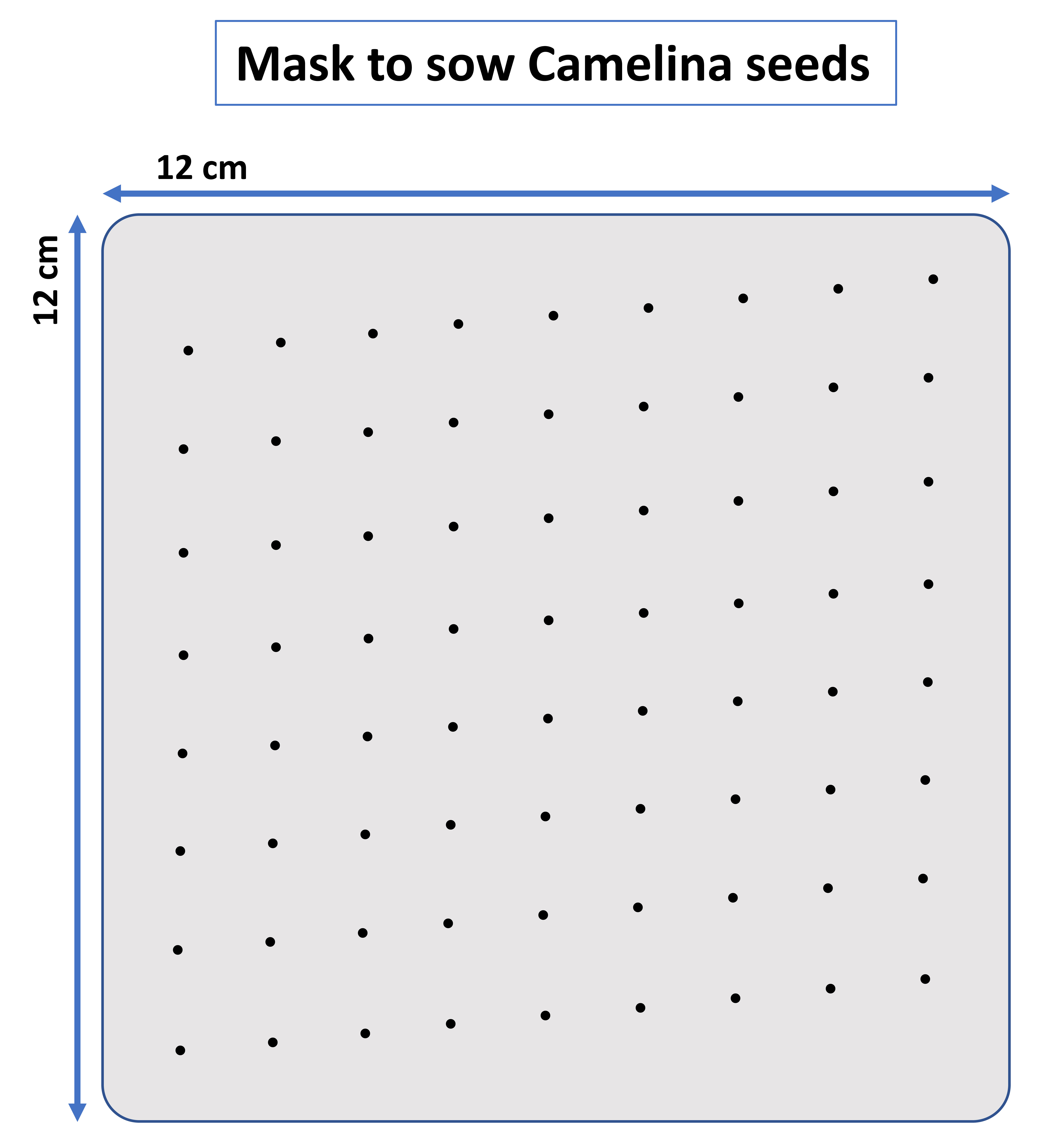

Supplement: Supplementary file 6 — Additional file 6. Supports that can be used to help for the seeds sowing on the biochemical assays for Camelina sativa. [file 13007_2018_377_MOESM6_ESM.jpg]

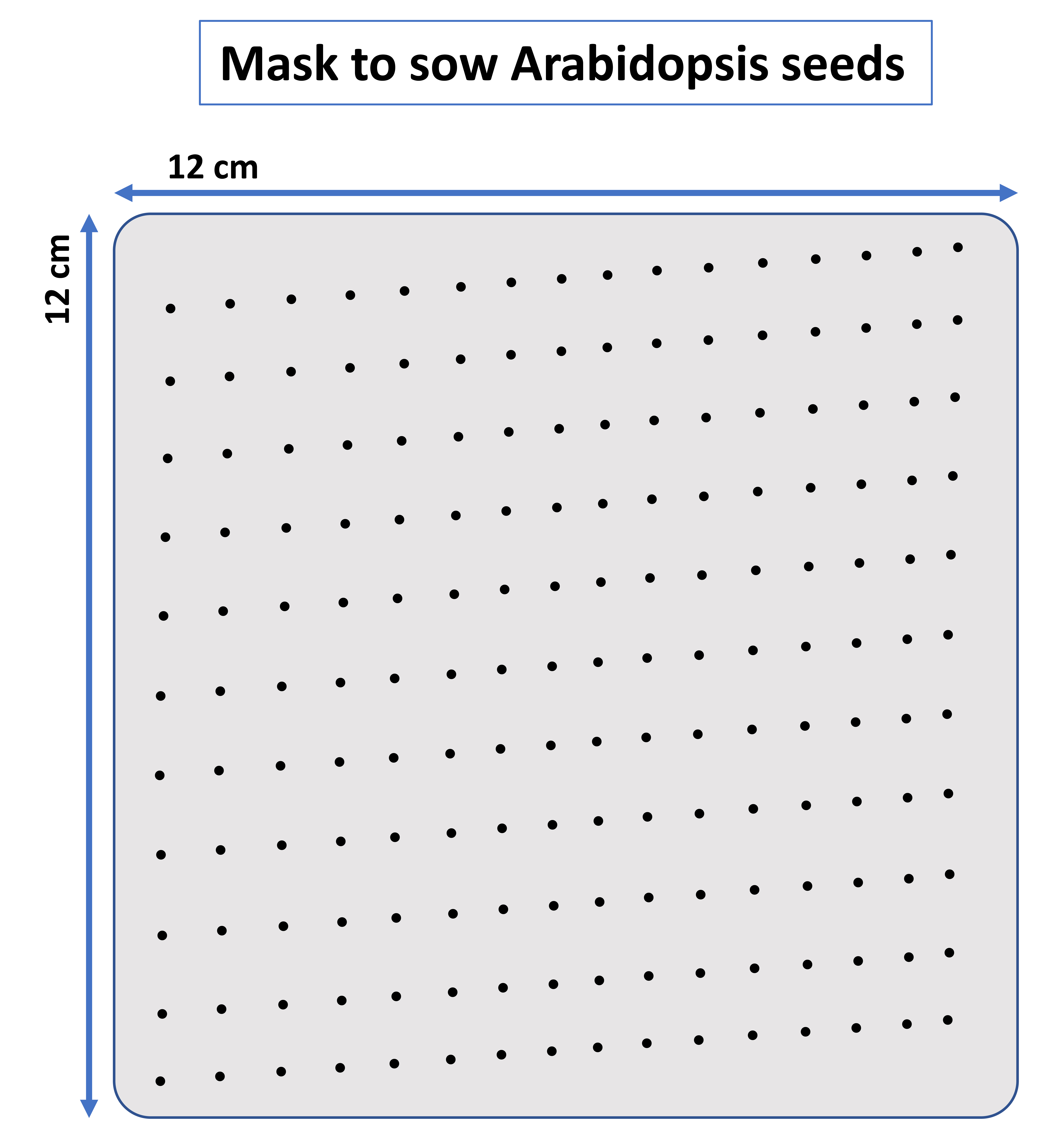

Supplement: Supplementary file 7 — Additional file 7. Supports that can be used to help for the seeds sowing on the biochemical assays for Arabidopsis thaliana. [file 13007_2018_377_MOESM7_ESM.jpg]

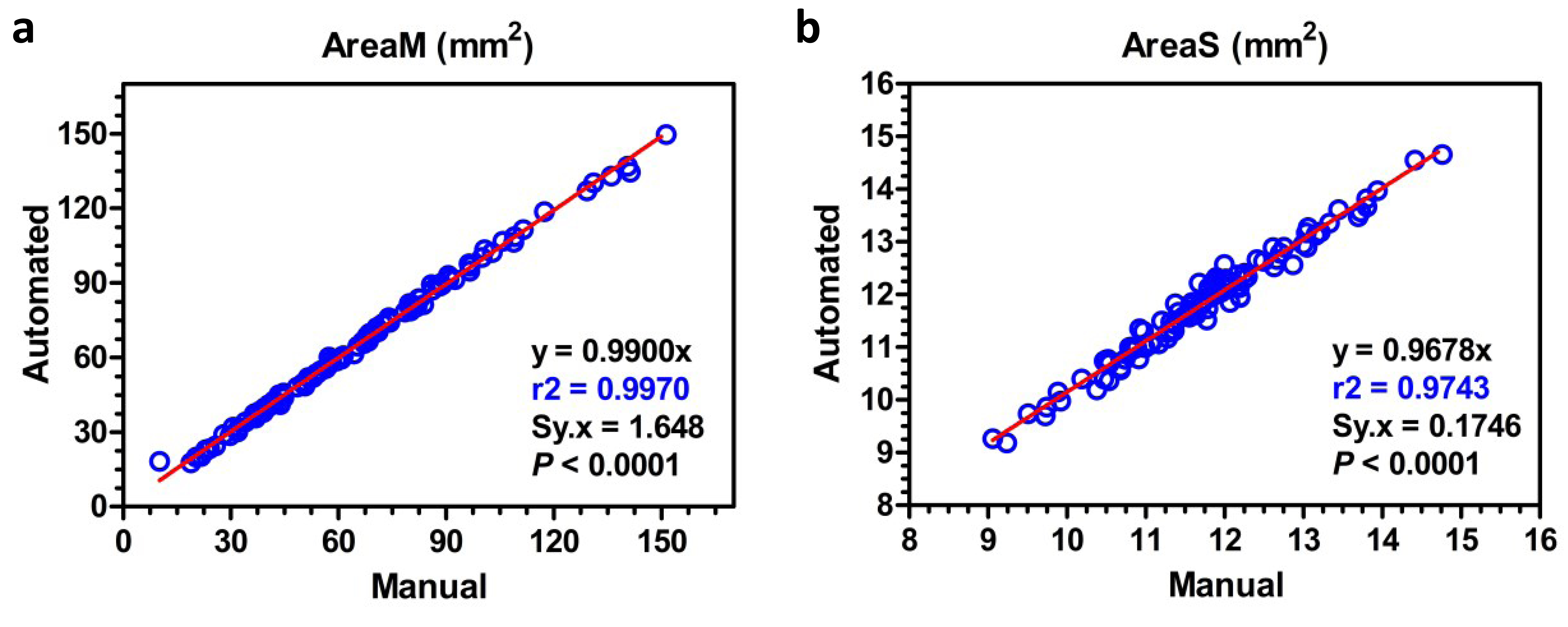

Supplement: Supplementary file 9 — Additional file 9. Correlation analysis of automated (MuSeeQ) versus manual (Fiji) measurements for (a) the area of the surface of soluble mucilage released in 2D (AreaM) and (b) the area of the seeds surface projected in 2D (AreaS). Since data are sampled from Gaussian populations (D’Agostino & Pearson omnibus normality test), Pearson’s correlation coefficients were determined by linear regression analyses on n = 104 seeds from twenty RILs and nine agarose stained gels. [file 13007_2018_377_MOESM9_ESM.jpeg]

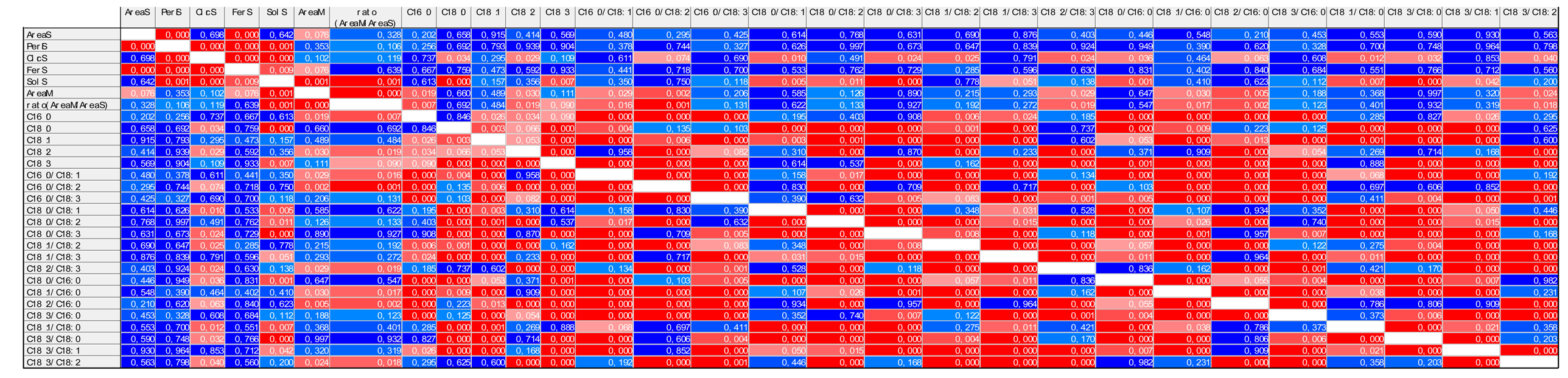

Supplement: Supplementary file 10 — Additional file 10. Pearson’s correlation matrix between the soluble mucilage, seed shape parameters and FAs-releated traits. The negative correlations are depict in red whereas positive correlations are in blue. The colour intensity follows the strength of the correlation. [file 13007_2018_377_MOESM10_ESM.jpeg]

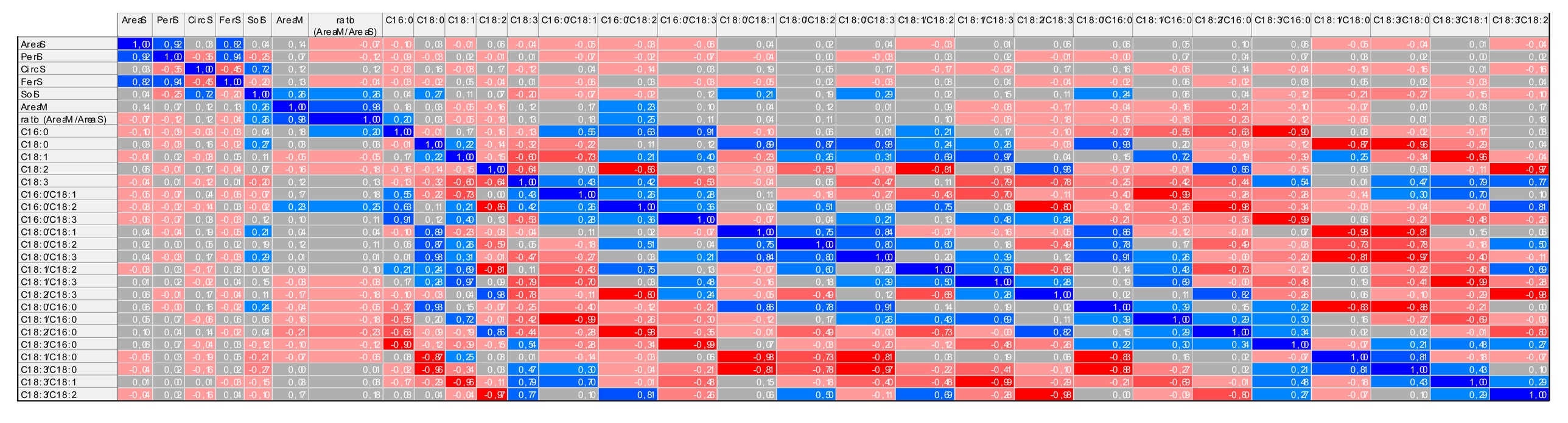

Supplement: Supplementary file 11 — Additional file 11. Likelihood matrix corresponding to the Pearson’s correlation matrix between the soluble mucilage, seed shape parameters and fatty acids-releated traits. The negative correlations are depict in red whereas positive correlations are in blue. The colour intensity follows the strength of the correlation. [file 13007_2018_377_MOESM11_ESM.jpeg]
