# Supplementary material for: MuSeeQ, a novel supervised image analysis tool for the simultaneous phenotyping of the soluble mucilage and seed morphometric parameters
Source: Plant Methods. 2018 Dec 18;14:112. doi: 10.1186/s13007-018-0377-5 (PMC6297999; doi:10.1186/s13007-018-0377-5)
Supplement: Supplementary file 8 — Additional file 8. The script of the MuSeeQ macro. [file 13007_2018_377_MOESM8_ESM.docx]

**// "MuSeeQ"**

**// This version of the tool MuSeeQ was especially designed for Linum usitatissimum L. seeds**

**// This macro segments seeds and soluble mucilages from input images and applies a specific number and color on them.**

**// Each seed and its respective mucilage are associated to the same number and the same color.**

**// Fabien Miart, 2018**

**requires("1.29i");**

**setTool(4);**

**waitForUser("Before both segmentations, please check the scale by selecting between both ends using the straigh tool")**

**run("Set Scale...");**

**setBatchMode(true);**

**rename("image1");**

**run("Duplicate...", "title=image2");**

**run("Duplicate...", "title=mucilage");**

**parameters();**

**run("Images to Stack", "name=Stack title=[] use keep");**

**selectWindow("seed");**

**close();**

**selectWindow("mucilage");**

**close();**

**selectWindow("image2");**

**close();**

**setBatchMode(false);**

**setTool(11);**

**waitForUser("Zoom on seeds to check both segmentations")**

**function parameters() {**

**width=512;**

**height=512;**

**Dialog.create("Set Threshold");**

**Dialog.addMessage("Mucilage Segmentation:");**

**Dialog.addNumber("Hue:", 172);**

**Dialog.addNumber("Brightness:", 85);**

**Dialog.addMessage("Seed Segmentation:");**

**Dialog.addNumber("Brightness:", 85);**

**Dialog.addHelp("http://rsb.info.nih.gov/ij/docs/");**

**Dialog.show();**

**X();**

**X2();**

**}**

**function X() {**

**Seg1 = Dialog.getNumber();**

**Seg2 = Dialog.getNumber();**

**min=newArray(3);**

**max=newArray(3);**

**filter=newArray(3);**

**a=getTitle();**

**selectWindow("image1");**

**run("HSB Stack");**

**run("Convert Stack to Images");**

**selectWindow("Hue");**

**rename("0");**

**selectWindow("Saturation");**

**rename("1");**

**selectWindow("Brightness");**

**rename("2");**

**min[0]=0;**

**max[0]= Seg1;**

**filter[0]="stop";**

**min[1]=24;**

**max[1]=255;**

**filter[1]="pass";**

**min[2]=0;**

**max[2]=Seg2;**

**filter[2]="stop";**

**for (i=0;i<3;i++){**

**selectWindow(""+i);**

**setThreshold(min[i], max[i]);**

**run("Convert to Mask");**

**if (filter[i]=="stop") run("Invert");**

**}**

**imageCalculator("AND create", "0","1");**

**imageCalculator("AND create", "Result of 0","2");**

**for (i=0;i<3;i++){**

**selectWindow(""+i);**

**close();**

**}**

**selectWindow("Result of 0");**

**close();**

**selectWindow("Result of Result of 0");**

**rename(a);**

**// Colour Thresholding-------------**

**run("Set Measurements...", "area perimeter shape feret's limit display add redirect=None decimal=8");**

**run("Analyze Particles...", "size=18-315 circularity=0.00-3.00 show=[Count Masks] display exclude clear include record add in_situ");**

**run("Summarize");**

**run("3-3-2 RGB");**

**run("From ROI Manager");**

**run("Labels...", "color=red font=36 show");**

**run("Overlay Options...", "stroke=white width=2 fill=none apply show");**

**roiManager("Show All with labels");**

**rename("x");**

**run("Flatten");**

**rename("seed");**

**selectWindow("x"); close();**

**IJ.renameResults("Mucilage_Results");**

**}**

**function X2() {**

**Seg3 = Dialog.getNumber();**

**min=newArray(3);**

**max=newArray(3);**

**filter=newArray(3);**

**b=getTitle();**

**selectWindow("mucilage");**

**run("HSB Stack");**

**run("Convert Stack to Images");**

**selectWindow("Hue");**

**rename("0");**

**selectWindow("Saturation");**

**rename("1");**

**selectWindow("Brightness");**

**rename("2");**

**min[0]=0;**

**max[0]=255;**

**filter[0]="pass";**

**min[1]=0;**

**max[1]=255;**

**filter[1]="pass";**

**min[2]=0;**

**max[2]= Seg3;**

**filter[2]="pass";**

**for (i=0;i<3;i++){**

**selectWindow(""+i);**

**setThreshold(min[i], max[i]);**

**run("Convert to Mask");**

**if (filter[i]=="stop") run("Invert");**

**}**

**imageCalculator("AND create", "0","1");**

**imageCalculator("AND create", "Result of 0","2");**

**for (i=0;i<3;i++){**

**selectWindow(""+i);**

**close();**

**}**

**selectWindow("Result of 0");**

**close();**

**selectWindow("Result of Result of 0");**

**rename(b);**

**// Colour Thresholding-------------**

**run("Set Measurements...", "area perimeter shape feret's limit display add redirect=None decimal=8");**

**run("Analyze Particles...", "size=8-15 circularity=0.00-3.00 show=[Count Masks] display exclude clear record add in_situ");**

**run("Summarize");**

**run("3-3-2 RGB");**

**run("From ROI Manager");**

**run("Labels...", "color=red font=30 show");**

**run("Overlay Options...", "stroke=white width=2 fill=none apply show");**

**roiManager("Show All with labels");**

**rename("x2");**

**run("Flatten");**

**rename("mucilage");**

**selectWindow("x2"); close();**

**IJ.renameResults("Seed_Results");**

**}**
