# Supplementary material for: MuSeeQ, a novel supervised image analysis tool for the simultaneous phenotyping of the soluble mucilage and seed morphometric parameters
Source: Plant Methods. 2018 Dec 18;14:112. doi: 10.1186/s13007-018-0377-5 (PMC6297999; doi:10.1186/s13007-018-0377-5)
Supplement: Supplementary file 12 — Additional file 12. Protocol for the preparation of the biochemical assay. [file 13007_2018_377_MOESM12_ESM.pdf]

Protocol for the preparation of the biochemical assay

First, note that the toluidine blue 0 dye and agarose medium must be prepared separately.

1. Prepare an 0,6% agarose medium (Sigma Aldrich) for 400 ml of pure water [With 400 ml, you can prepare 14 or 15 square petri dishes].
2. Then, prepare 10 mL of 0,4% toluidine blue 0 (Sigma Aldrich) dye solution in a tube of 15 ml.
3. Add 0,01mg of Sodium Tetraborate anhydrous (Sigma Aldrich).
4. Add 10 µl of Tween 20.
5. Vortex strongly.
6. Heat up the agarose medium in a microwave [until boiling; approximately 3 min], mix and repeat until boiling.
7. Wait for 30 min that the medium cool down.
8. Add 4 ml of 0,4% toluidine blue 0 in the 400 ml of agarose medium and mix in the hand.
9. Measure 50 ml and pour the medium in the square petri dishes (120*120*17 mm) [the surface of the bench must be perfectly flat].
10. Wait at least 1 hour for the medium air drying, but no more than 4 hours to sow the seeds.
